# Improving the accuracy of estimates of the pulse sequence period using the methodology of complete sufficient statistics

**DOI:** 10.1038/s41598-022-24457-2

**Published:** 2022-11-19

**Authors:** Konstantin Zhuchkov, Mikhail Vasilchenko, Anna Zagrebneva, Alexey Zavyalov

**Affiliations:** 1 National University of Oil and Gas “Gubkin University”, 65, Bld. 1 Leninsky Prospekt, Moscow, Russia 119991; 2 School of Engineering, RMIT University, GPO Box 2476, Melbourne, VIC 3001 Australia; 3 Faculty of IT Systems and Technologies, Don State Technical University, Gagarin Sqr.1, Rostov-On-Don, Russia 344000

**Keywords:** Design, synthesis and processing, Information technology, Statistics

## Abstract

This paper is devoted to the synthesis of new signal processing algorithms based on the methodology of complete sufficient statistics and the possibility of using the Lehmann–Scheffe theorem. Using the example of a sequence of quasi-rectangular pulses, an approach to estimating their period was illustrated, taking into account the duty-off factor and the pulse squareness coefficient. A mathematical model was developed, on the basis of which, estimates of the potential accuracy of the methods were carried out. It is established that for the sample size value (n > 8), the relative root-mean-square error of estimating the repetition period using the methodology of complete sufficient statistics is lower than that of the traditional estimate. In addition to theoretical calculations, simulation results confirming the achieved effect are presented. The results obtained have a wide range of applicability and can be used in the design of control and measuring equipment in the oil and gas industry, in the development of medical equipment, in the field of telecommunications, in the design of pulse-Doppler radars, etc.

## Introduction

The development of microelectronic components and computing platforms in the rapidly developing modern world creates new challenges for the development of algorithms and new approaches in the processing of real-time information. The synthesis of information processing algorithms has to be carried out in the presence of a complex set of interfering factors: temporal and spatial heterogeneity of the characteristics of useful signals and interference, sensitivity heterogeneity and sensor defects, pulse interference, etc. Even if all the interfering factors could be fully taken into account, the signal and noise environment model would be very complex, and signal processing algorithms based on such a model would be unrealizable in real time. In addition, these factors are inherently random and there may be a priori uncertainty in the description of real signals. Thus, the synthesis of algorithms for estimating signal parameters, as a rule, has to be carried out under conditions of multiple of interfering factors and a priori uncertainty.

In the condition of uncertainty in the initial data, attempts are made to solve the problem of evaluating the efficiency of the algorithm for measuring signal parameters against the background of noise using the classical Bayesian criterion^1^. However, the complexity of these attempts is associated with the requirement for a priori probabilities of the presence and absence of a signal in the observed sample and risk estimation.

At the same time, there is a growing demand for the synthesis of new algorithms for solving problems of estimating signal parameters in a wide range of applications: radars^2^, communication systems^3,4^, electronic intelligence^5^, X-Ray absorption spectra^6^, Bragg scattering^7^, plasma parameters estimation^8,9^, assessment of the stability of information systems^10^, the solution of the problem of parameter estimation for in-tube flaw detection^11^, the development of optoelectronic displacement sensors^12^, etc. The requirements for the accuracy of the assessment and its reliability are increasing, which determines both the use of more productive computing resources and determines the relevance of the search for new information processing algorithms.

The purpose of this work is an attempt to synthesize an algorithm for estimating the period of quasi-rectangular pulses under conditions of possible loss of a part of the sequence. The construction of a signal model was also part of this work. The novelty of the problem statement and the above solution concludes in the use of methods of complete sufficient statistics based on the application of the Rao–Blackwell estimate for the Lehman–Scheffe theorem in the synthesis of the estimation algorithm^13,14^. If the conditioning statistics are complete and sufficient, and the initial estimate is unbiased, then the Rao-Blackwell estimate is the best unbiased estimate^15^.

At the same time, it is well known^16^ that Gaussian noise maximum likelihood estimation (MLE) is asymptotically optimal, i.e., the stability of its operations and the efficiency of the estimates are achieved at high signal/noise ratios and an infinite sample size^17^. In the context of the problems of estimating the parameters of real signals, there is a need to search for algorithms that can work no worse with finite samples and low signal/noise ratios. That way, for example, in order to perform the parameter estimation for the sine combination signals and periodic signals the multi-innovation stochastic gradient algorithm based on the gradient optimization principle is presented in^18^. A closed-form maximum likelihood amplitude estimator is obtained in^19^. The extended noise-resistant correlation method to identify the period of pseudo-periodic signals is derived in^20^. Attempts are being made to use Fourier and wavelet transformation with windows of various kinds to increase the localization of signal energy^21,22^ or multi-threshold circuits^23,24^ are used, which give a gain in the accuracy of the estimate in particular cases. However, in general, they carry the risk of instability proposed algorithm. It is also possible to use neural network methods to solve the problem of estimating signal parameters, but the main disadvantage here is a multiple increase in the sample size for training^25^, even with a slight change to the signal–noise environment.

An important point for solving this problem is to ensure the stability of the proposed algorithm under conditions of a high level of a priori uncertainty. Also, when synthesizing an algorithm, it is necessary to establish the complexity class and estimate the runtime realizability limitations in the context of current computing hardware capabilities.

In radio engineering applications, the distributions of samples and observed technical processes often have complete sufficient statistics, which creates the prerequisites for the application of the Lehmann–Scheffe theorem in finding effective estimates of signal parameters. The application of the estimates obtained can find a place not only in traditional problems of estimating the repetition period, but also in several problems that reduce the problem of finding the repetition period. Two examples are: when implementing a clock recovery circuit for a demodulator operating on short signal samples^26^, and when the phase-locked loop capture time is significantly longer than the signal sample duration^27^.

## Mathematical signal model

Consider the process $x\left( t \right)$ on the segment $\left[ {0,\Delta T} \right]$, which is an additive mixture of signal and noise:

$$x\left( t \right) = A_{m} s\left( t \right) + \eta \left( t \right)$$

where $A_{m}$ is the signal amplitude, $s\left( t \right)$ is the normalized signal, $\eta \left( t \right)$ is the differentiable stationary Gaussian noise characterized by the variance $\sigma^{2}$ and the normalized correlation function *r(t)*. The distribution of the energy parameters of the signal and noise will be characterized by the signal/noise ratio *q* = *A*_*m*_*/σ*.

**Figure 1:**
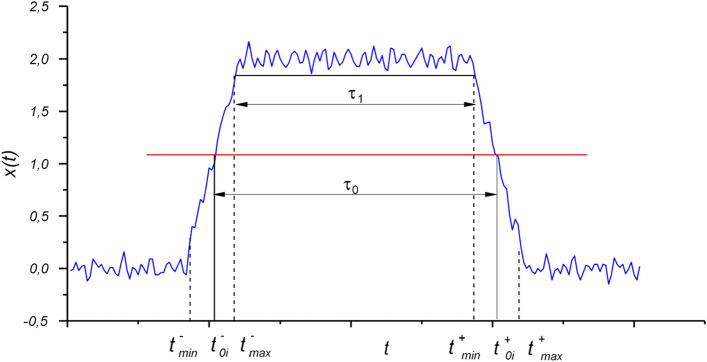
Observed process x(t) in the form of the impulse on the noise background.

Let the leading edges of the signal pulse $s\left( t \right)$ intersect the level *H* at time $t_{0i}^{ + }$, and the trailing at time $t_{0i}^{ - }$:

1 $$\begin{array}{*{20}l} {t_{0i}^{ + } = t_{00}^{ + } + iT,} \hfill & {i = 0,1, \ldots } \hfill \\ {t_{0i}^{ - } = t_{00}^{ + } + iT + {T \mathord{\left/ {\vphantom {T {v,}}} \right. \kern-\nulldelimiterspace} {v,}}} \hfill & {i = 0,1, \ldots ,} \hfill \\ \end{array}$$

where *T* is the signal period; *ν* is the off-duty factor; $t_{00}^{ + }$ is the first crossing of *H* level. Then, for large signal/noise ratios, q >> 1, the process *x(t)* will cross the *H* level at times

2 $$t_{i}^{ + } = t_{0i}^{ + } + \Delta t_{0i}^{ + } ,\quad t_{i}^{ - } = t_{0i}^{ - } + \Delta t_{0i}^{ - } ,$$

where $\Delta t_{0i}^{ + }$, $\Delta t_{0i}^{ - }$ are small random additions, due to the effect of noise *η(t)*. Figure 1 schematically shows the process and the designations adopted in the work.

It is assumed that the pulses have a quasi-rectangular shape and can be approximated according to^28^ by the following function

3 $$s(x) = \left\{ {\begin{array}{*{20}l} {\exp \left\{ { - \frac{\pi }{8}\frac{{\left( {2xk - 1} \right)^{2} }}{{\left( {k - 1} \right)^{2} }}} \right\},} \hfill & {x > \frac{1}{2k},} \hfill \\ {1\,,} \hfill & {\left| x \right| \le \frac{1}{2k},} \hfill \\ {\exp \left\{ { - \frac{\pi }{8}\frac{{\left( {2xk + 1} \right)^{2} }}{{\left( {k - 1} \right)^{2} }}} \right\},} \hfill & {x < - \frac{1}{2k}.} \hfill \\ \end{array} } \right.$$

Here $k$ is the coefficient of the pulse squareness, it equals the ratio of pulse duration at the intersection of H level $\tau_{0}$ to the pulse duration $\tau_{1}$, $k = \tau_{0} /\tau_{1}$ (see Fig. 1).

The objective of this research is to synthesize the most effective estimation of the repetition period of quasi-rectangular pulses against background noise and interference, under conditions of a priori uncertainty of its repeatability (period and phase). An additional complication of the model is the possible loss of a pulse in the sequence according to a pseudo-random law, which is not known a priori. The known parameters are: off-duty factor (excluding loss), squareness coefficient and the times when the signal leading and trailing edges cross the H level.

## Methodology

### Description of maximum likelihood estimation method

The measurement process of the pulse repetition period is derived from the process of measuring the time of the beginning (end) of the pulse. Taking into account that the parameter (classical in the theory of maximum likelihood estimates) is non-energetic, in estimating the lower bound of the accuracy of pulse repetition period measurement, we take as a basis the expression for the elements of the Fisher matrix:

4 $$\Phi_{ik} = - q^{2} \frac{{\partial^{2} \Psi (\lambda )}}{{\partial \lambda_{i} \partial \lambda_{k} }}\left| {_{\lambda = 0} } \right.$$

where $\Psi (\lambda )$ is the uncertainty function of the vector $\lambda$. Since the inverse Fisher matrix is the correlation matrix of the MLE of all simultaneously estimated parameters^29^, and the dispersions of the MLE of these parameters are on the main diagonal of this matrix, and taking into account that the focus is only on one non-energy parameter – the pulse arrival time ($\tilde{t}$), it can be written as:

5 $$D\left\{ {\hat{\tilde{t}}|\tilde{t}} \right\} = - 1/q^{2} \Psi^{\prime\prime}(0)$$

where $\hat{\tilde{t}}$ is the MLE of the pulse arrival time. Assuming that the uncertainty function is stationary with respect to the parameter $t - \tilde{t}$ it gives the following expression:

6 $$\Psi (t - \tilde{t}) = \frac{1}{2E}\int\limits_{ - \infty }^{\infty } {s(t - \tilde{t}} )s^{*} (t)dt$$

where *E* is a signal energy. After applying Parseval theorem and double differentiation (3) dispersion of MLE for the parameter $\tilde{t}$ is bounded by the expression below:

7 $$D\left\{ {\hat{\tilde{t}}|\tilde{t}} \right\} = \frac{{\int\limits_{ - \infty }^{\infty } {|S(f)|^{2} df} }}{{4\pi^{2} q^{2} \int\limits_{ - \infty }^{\infty } {f^{2} |S(f)|^{2} df} }}$$

where $S\left( f \right)$ is the Fourier transformation of the complex envelop of the signal $s\left( t \right)$. Taking into account the approximation in which the pulse is rectangular^30^ with duration $\tau_{0}$ (passed through a perfect filter with passband ∆f, where ∆f $\tau_{0}$≈1), the following assumption can be used (S(f) = |sin(πf$\tau_{0}$)|/f$\tau_{0}$ and normalization condition for *S(f)*^14^):

8 $$\frac{{\int\limits_{ - \infty }^{\infty } {f^{2} |S(f)|^{2} df} }}{{\int\limits_{ - \infty }^{\infty } {|S(f)|^{2} df} }} \approx \frac{1}{{\tau_{0}^{2} }}.$$

In this case, if a sequence of pulses with an off-duty factor $\nu = T/\tau_{0} = 2$ (square wave) is an input of the meter, the lower boundary of the estimated variance of the arrival timefor each pulse as defined by Eq. (5), is presented as:

9 $$D\left\{ {\hat{\tilde{t}}|\tilde{t}} \right\} = \frac{{T^{2} }}{{16\pi^{2} q^{2} }}$$

The most common method for estimating the period is based on measuring the difference between the start times of adjacent pulses. If the assessment is made by $n$ adjacent periods, then its relative error decreases $n$ times

10 $$\delta_{0} = \frac{{\sqrt {D\left\{ {\hat{\tilde{t}}|\tilde{t}} \right\}} }}{nT} = \frac{1}{4\pi qn}$$

If the period is estimated using the information contained in the statistics of the pulse end times (crossing threshold by the trailing edge), then the estimate can be improved by $\sqrt 2$. Thus, we can assume that for traditional estimates the video pulse repetition with an off duty factor = 2, the relative root-mean-square (RMS) error is

11 $$\delta_{0} = \frac{1}{4\sqrt 2 \pi qn}$$

The estimate (11) is the asymptotic accuracy bound for the method (the limiting value of the Cramer-Rao inequality^16^).

An example of the traditional estimation of the pulse repetition period by the MLE method is the expression:

12 $$\tilde{T} = \frac{1}{2n}\sum\limits_{k = 1}^{n} {\left( {\frac{{t_{k}^{ + } - t_{k - 1}^{ + } + t_{k}^{ - } - t_{k - 1}^{ - } }}{{i_{k} - i_{k - 1} }}} \right)}$$

where, $t_{k}^{ + }$, $t_{k}^{ - }$ are the times of intersection of the leading and trailing pulse edges (10), and $i_{k}$ is the number of the pulse in the sequence. When determining the numbers $i_{k}$, all pulses are considered, even those that were missed and did not fall into the sample.

### Description of complete sufficient statistics estimation method

Consider the statistics ***t***^**+**^  = *{*${t}_{0}^{+}$*, … ,*${t}_{n}^{+}$*}* and ***t***^***–***^ = *{*${t}_{0}^{-}$*,…, *${t}_{n}^{-}$*}*, where $t_{i}^{ + }$ and $t_{i}^{ - }$ are given (2) to synthesize the estimate of the period *T*. To find the joint probability density of the statistics *t*^+^ and *t*^*-*^, the results of^13–15,26,27^ are used. Suppose that with a probability close to one, the crossing of the level *H* will occur in disjointed bounds (${t}_{i{\text{min}}}^{+},{t}_{i{\text{max}}}^{+}$) and (${t}_{i{\text{min}}}^{-},{t}_{i{\text{ma}}{\text{x}}}^{-}$) at points $t_{0i}^{ + }$ and $t_{0i}^{ - }$ (Fig. 1). If the probabilities of false crossings of the level *H* are small and the condition below is satisfied^23^:

13 $$\Phi \left( {\frac{{U_{m} \left| {s^{\prime}(t_{0i}^{ - } )} \right|}}{{\sigma_{1} }}} \right) + \frac{{\sigma_{1} }}{{U_{m} \left| {s^{\prime}(t_{0i}^{ - } )} \right|\sqrt {2\pi } }}\exp \left\{ {\frac{{U_{m}^{2} \left| {s^{\prime}(t_{0i}^{ - } )} \right|^{2} }}{{\sigma_{1}^{2} }}} \right\} \approx 1$$

$$\Phi \left( x \right) = \frac{1}{{\sqrt {2\pi } }}\int\limits_{ - \infty }^{x} {e^{{ - \frac{{t^{2} }}{2}}} dt}$$

where $\Phi \left( x \right)$ is the Laplace integral, $\sigma_{1}^{2} = - \sigma \,r^{\prime\prime}(0)$ is the variance of the derivative of the process *η(t)*, and *r''(0)* is the value of the second derivative of the normalized correlation function *r(t)* at *t* = *0*, then the one dimensional probability distribution densities at level *H* is as follows^14^:

14 $$\begin{gathered} \rho (t_{i}^{ + } ) = \frac{1}{{\sqrt {2\pi \sigma_{ + }^{2} } }}\,\exp \left\{ { - \frac{{(t_{i}^{ + } - t_{oo}^{ + } - iT)^{2} }}{{2\sigma_{ + }^{2} }}} \right\}, \hfill \\ \rho (t_{i}^{ - } ) = \frac{1}{{\sqrt {2\pi \sigma_{ - }^{2} } }}\exp \,\left\{ { - \frac{{(t_{i}^{ - } - t_{oo}^{ + } - {T \mathord{\left/ {\vphantom {T v}} \right. \kern-\nulldelimiterspace} v} - iT)^{2} }}{{2\sigma_{ - }^{2} }}} \right\}, \hfill \\ \end{gathered}$$

where $$\sigma_{ + }$$ = *1/[q |s'(*$${t}_{0i}^{+}$$*)|],* $$\sigma_{ - }$$ = *1/[q |s'(*$${t}_{0i}^{-}$$*)|]*.

Then, under the assumption that the processes within the intervals of the leading and trailing edges of the pulse will be statistically independent, the joint probability density distribution is obtained for the sample vectors **t**^**+**^ и **t**^**–**^:

15 $$\rho ({\mathbf{t}}^{ + } ,{\mathbf{t}}^{ - } ) = \prod\limits_{i = 0}^{n} {\rho (t_{i}^{ + } )\rho (t_{i}^{ - } )} = \left( {\frac{1}{{2\pi \sigma_{ + } \sigma_{ - } }}} \right)^{n + 1} \times \exp \left\{ { - \sum\limits_{i = 0}^{n} {\frac{{(t_{i}^{ + } - t_{oo}^{ + } - iT)^{2} }}{{2\sigma_{ + }^{2} }}} - \sum\limits_{i = 0}^{n} {\frac{{(t_{i}^{ - } - t_{oo}^{ + } - {T \mathord{\left/ {\vphantom {T v}} \right. \kern-\nulldelimiterspace} v} - iT)^{2} }}{{2\sigma_{ - }^{2} }}} } \right\}.$$

Then we find an effective estimate of the pulse repetition period, assuming that the moduli of the slope of the leading and trailing edges of the signal coincide, *|s'(*${t}_{0i}^{+}$*)|* = *|s'(*${t}_{0i}^{-}$*)|* (i.e. parameters $\sigma_{ + }$ and $\sigma_{ - }$ are equal). We assume that the off-duty factor ν and signal to noise ratio *q are known*, and that the value of the modulus of the front slope *|s'(*${t}_{0i}^{-}$*)| of* the initial signal phase is not a priori determined.

The solution of this problem using the methodology of CSS is based on the theorem of the uniqueness of an effective estimate^29^, which is a consequence of the Lehmann–Scheffe theorem^13^.

According to the uniqueness theorem for the effective estimate, if the estimated signal characteristic parameter µ can be presented as a function depending on the parameter ${\mathbf{Y}} = \left\{ {Y_{1} ,\,Y_{2} , \ldots Y_{m} } \right\}$, where ${\mathbf{Y}}$ is a vector of expansion of the joint probability distribution density $\rho ({\mathbf{t}}^{ + } ,{\mathbf{t}}^{ - } )$:

16 $$\mu = g({\mathbf{Y}}) = \sum\limits_{i = 1}^{M} {\sum\limits_{j = 0}^{N} {a_{i,j} m_{i}^{j} ({\mathbf{Y}})} } ,\quad - \infty < a_{i,j} < + \infty$$

Then the only effective estimate of the parameter µ is given^13,29^ by the expression:

17 $$\mu_{^{\prime}}^{*} = \sum\limits_{i = 1}^{M} {\sum\limits_{j = 0}^{N} {a_{i,j} Y_{i}^{j} } }$$

Here, *M* is the dimension of the complete sufficient statistics ${\mathbf{Y}} = \left\{ {Y_{1} ,\,Y_{2} , \ldots Y_{m} } \right\}$*, N* is the maximum number of the moment order, $m_{i}^{j} ({\mathbf{Y}}) = M\left\{ {Y_{i}^{j} } \right\}$*, i* = *1,…,M; j* = *0,…,N*; $m_{i}^{0} ({\mathbf{Y}}) = 1.$

Let 's define complete sufficient statistics ${\mathbf{Y}} = \left\{ {Y_{1} ,\,Y_{2} , \ldots Y_{m} } \right\}$. Considering the conditions of the problem ($\sigma_{ + } = \sigma_{ - }$), the joint probability distribution density $\rho ({\mathbf{t}}^{ + } ,{\mathbf{t}}^{ - } )$ (15) has the following form:

18 $$\rho ({\mathbf{t}}^{ + } ,{\mathbf{t}}^{ - } ) = \left( {\frac{1}{{2\pi \sigma_{ + }^{2} }}} \right)^{n + 1} \times \exp \left\{ { - \sum\limits_{i = 0}^{n} {\frac{{(t_{i}^{ + } - t_{oo}^{ + } - iT)^{2} }}{{2\sigma_{ + }^{2} }}} - \sum\limits_{i = 0}^{n} {\frac{{(t_{i}^{ - } - t_{oo}^{ + } - {T \mathord{\left/ {\vphantom {T v}} \right. \kern-\nulldelimiterspace} v} - iT)^{2} }}{{2\sigma_{ + }^{2} }}} } \right\}.$$

A practical way to find sufficient statistics ${\mathbf{Y}}\left( x \right)$ is to use the factorization theorem^29,31,32^, according to which the conditional probability density distribution $\rho_{{{\varvec{\uptheta}}}} (x) = P(X = x|{{\varvec{\uptheta}}})$ must be represented as:

$$\rho_{{{\varvec{\uptheta}}}} (x) = h\left( x \right)g\left( {{{\varvec{\uptheta}}},{\mathbf{Y}}\left( x \right)} \right)$$

The resulting expression for ${\mathbf{Y}}\left( x \right)$ is a sufficient statistic.

We factorize the conditional probability density $\rho ({\mathbf{t}}^{ + } ,{\mathbf{t}}^{ - } )$ (18) for fixed parameters $\sigma_{ + }$, $t_{00}^{ + }$, $T$. We denote:

19 $${{\varvec{\uptheta}}} = \left\{ {\vartheta_{1} ,\,\vartheta_{2} ,\,\vartheta_{3} } \right\} = \left\{ {\frac{{t_{00}^{ + } }}{{\sigma_{ + }^{2} }},\,\frac{T}{{\sigma_{ + }^{2} }}_{2} , - \frac{1}{{\sigma_{ + }^{2} }}} \right\}$$

Considering the first sum in (18) and collecting the terms containing random variables with the parameters *ϑ*_*1*_*, ϑ*_*2,*_* ϑ*_*3*_, the remaining terms are assigned to the function *C*_*1*_*(ϑ*_*1*_*, ϑ*_*2,*_* ϑ*_*3*_*)*:

$$\begin{aligned} - \sum\limits_{i = 0}^{n} {\frac{{(t_{i}^{ + } - t_{oo}^{ + } - iT)^{2} }}{{2\sigma_{ + }^{2} }}} & = - \frac{1}{{2\sigma_{ + }^{2} }}\sum\limits_{i = 0}^{n} {\left( {t_{i}^{ + } } \right)^{2} - 2t_{oo}^{ + } t_{i}^{ + } - 2iTt_{i}^{ + } + 2iTt_{oo}^{ + } + \left( {iT} \right)^{2} + } \left( {t_{oo}^{ + } } \right)^{2} \\ & = - \frac{1}{{2\sigma_{ + }^{2} }}\sum\limits_{i = 0}^{n} {\left( {t_{i}^{ + } } \right)^{2} + } \frac{{t_{oo}^{ + } }}{{\sigma_{ + }^{2} }}\sum\limits_{i = 0}^{n} {t_{i}^{ + } } + \frac{T}{{\sigma_{ + }^{2} }}\sum\limits_{i = 0}^{n} {t_{i}^{ + } i} - \frac{{t_{oo}^{ + } }}{{\sigma_{ + }^{2} }}T\frac{n(n + 1)}{2} - \frac{{\left( {t_{oo}^{ + } } \right)^{2} (n + 1)}}{{2\sigma_{ + }^{2} }} - \frac{{T^{2} }}{{2\sigma_{ + }^{2} }}\frac{n\,(n + 1)(2n + 1)}{6} \\ & = \vartheta_{1} \sum\limits_{i = 0}^{n} {t_{i}^{ + } } + \vartheta_{2} \sum\limits_{i = 0}^{n} {t_{i}^{ + } i} + \vartheta_{3} \sum\limits_{i = 0}^{n} {\left( {t_{i}^{ + } } \right)^{2} + C_{1} \left( {\vartheta_{1} ,\vartheta_{2} ,\vartheta_{3} } \right)} , \\ \end{aligned}$$

20 $$C_{2} \left( {\vartheta_{1} ,\vartheta_{2} ,\vartheta_{3} } \right) = \frac{{(n + 1)\vartheta_{1}^{2} }}{{4\vartheta_{3} }} + \frac{{(n + 1)\vartheta_{1} \vartheta_{2} }}{{2\vartheta_{3} }} + \frac{{\vartheta_{2}^{2} }}{{4\vartheta_{3} }}\frac{n(n + 1)(2n + 1)}{6}$$

Similarly, an expression for another sum (18) gets:

$$- \sum\limits_{i = 0}^{n} {\frac{{(t_{i}^{ + } - t_{oo}^{ + } - iT)^{2} }}{{2\sigma_{ + }^{2} }}} = \vartheta_{1} \sum\limits_{i = 0}^{n} {t_{i}^{ - } } + \vartheta_{2} \sum\limits_{i = 0}^{n} {t_{i}^{ - } \left( {i + {1 \mathord{\left/ {\vphantom {1 \nu }} \right. \kern-\nulldelimiterspace} \nu }} \right)} + \vartheta_{3} \sum\limits_{i = 0}^{n} {\left( {t_{i}^{ - } } \right)^{2} + C_{2} \left( {\vartheta_{1} ,\vartheta_{2} ,\vartheta_{3} } \right)} ,$$

21 $$C_{2} \left( {\vartheta_{1} ,\vartheta_{2} ,\vartheta_{3} } \right) = \frac{{(n + 1)\vartheta_{1}^{2} }}{{4\vartheta_{3} }} + \frac{{(n + 1)\vartheta_{1} \vartheta_{2} }}{{2\nu \vartheta_{3} }} + \frac{{\vartheta_{2}^{2} }}{{4\vartheta_{3} }}\frac{(n + 1)(n\nu + 1)}{{\nu^{2} }}$$

After factorization, the probability density distribution $\rho ({\mathbf{t}}^{ + } ,{\mathbf{t}}^{ - } )$ (18), taking into account (19) and the transformations performed above, takes the form

22 $$\rho ({\mathbf{t}}^{ + } ,{\mathbf{t}}^{ - } ) = C\left( {{\varvec{\uptheta}}} \right)\,\exp \;\left\{ {\vartheta_{1} Y_{1} + \vartheta_{2} Y_{2} + \vartheta_{3} Y_{3} } \right\}$$

where

23 $$Y_{1} = \sum\limits_{i = 0}^{n} {\left( {t_{i}^{ + } + t_{i}^{ - } } \right)\,;} \quad Y_{2} = \sum\limits_{i = 0}^{n} {\left[ {it_{i}^{ + } + (i + {1 \mathord{\left/ {\vphantom {1 \nu }} \right. \kern-\nulldelimiterspace} \nu })t_{i}^{ - } } \right]\,;} \quad Y_{3} = \sum\limits_{i = 0}^{n} {\left[ {\left( {t_{i}^{ + } } \right)^{2} + \left( {t_{i}^{ - } } \right)^{2} } \right]\,;}$$

24 $$C({{\varvec{\uptheta}}}) = \left( { - \frac{{\vartheta_{3} }}{\pi }} \right)^{n + 1} \exp \left\{ {\frac{{(n + 1)\vartheta_{1}^{2} }}{{2\vartheta_{3} }} + \frac{{(n + 1)(n + {1 \mathord{\left/ {\vphantom {1 \nu }} \right. \kern-\nulldelimiterspace} \nu })\vartheta_{1} \vartheta_{2} }}{{2\vartheta_{3} }} + \frac{{\vartheta_{2}^{2} }}{{4\vartheta_{3} }}\left[ {\frac{n(n + 1)(2n + 1)}{6} + \frac{(n + 1)(n\nu + 1)}{{\nu^{2} }}} \right]} \right\}$$

Here the parameter *C(*θ*)* is obtained as *C(*θ*)* = *(− ϑ*_*3*_*/π) *^*n*+*1*^*exp{C*_*1*_*(*θ*)* + *C*_*2*_*(*θ*)}*, where *C*_*1*_*(*θ*)* and *C*_*2*_*(*θ*)* are represented by formulas (20) and (21). Thus, the conditional probability density $\rho ({\mathbf{t}}^{ + } ,{\mathbf{t}}^{ - } )$ takes the form:

$$\rho ({\mathbf{t}}^{ + } ,{\mathbf{t}}^{ - } ) = h\left( {{\mathbf{t}}^{ + } ,{\mathbf{t}}^{ - } } \right)g\left( {{{\varvec{\uptheta}}},{\mathbf{Y}}\left( {{\mathbf{t}}^{ + } ,{\mathbf{t}}^{ - } } \right)} \right)$$

where $h\left( {{\mathbf{t}}^{ + } ,{\mathbf{t}}^{ - } } \right) \equiv 1$ and $g\left( {{{\varvec{\uptheta}}},{\mathbf{Y}}} \right) = C\left( {{\varvec{\uptheta}}} \right)\,\exp \;\left\{ {{\mathbf{Y}} \cdot {{\varvec{\uptheta}}}} \right\}$ and according to the factorization theorem^29,31,32^, the statistics ${\mathbf{Y}}$ expressed by formula (23) are sufficient statistics.

Distribution density $\rho ({\mathbf{t}}^{ + } ,{\mathbf{t}}^{ - } )$ belongs to the exponential distribution with sufficient statistics **Y** = {*Y*_*1*_*, Y*_*2*_*, Y*_*3*_} and parameter **θ**. For unknowns, $t_{oo}^{ + }$*, T,* $\sigma_{ + }$ the parameter takes values from the region (0, ∞) × (0, ∞) × (− ∞, 0). We use the completeness theorem^27^, according to which if the probability distribution density belongs to the class of exponential distributions and the set of parameter values contains an *n*-dimensional interval, then the statistics **Y** is complete^29^. Then we can apply the theorem on the uniqueness of the effective estimate.

Let us express the period in terms of the mathematical expectation of the complete sufficient statistic **Y**. We calculate the mathematical expectation of *M*(*Y*_*1*_) and *M*(*Y*_*2*_), and obtain the system of equations for $t_{00}^{ + }$, *T*, ν. We get expressions for *Y*_*1*_ and *Y*_*2*_, giving similar ones:

25 $$\begin{gathered} Y_{1} = \sum\limits_{i = 0}^{n} {\left( {\Delta \,t_{0i}^{ + } + \Delta \,t_{0i}^{ - } } \right)} + 2(n + 1)t_{00}^{ + } + \left( {n + 1} \right)\left( {n + \frac{1}{\nu }} \right)T, \hfill \\ Y_{2} = \sum\limits_{i = 0}^{n} {\left( {\Delta \,t_{0i}^{ + } i + \Delta \,t_{0i}^{ - } \left( {i + \frac{1}{\nu }} \right)} \right)} + \left( {n + 1} \right)\left( {n + \frac{1}{\nu }} \right)\,t_{00}^{ + } + \left( {n + 1} \right)\left( {\frac{{3nv + v^{2} n(2n + 1) + 3}}{{3\nu^{3} }}} \right)\,T. \hfill \\ \end{gathered}$$

Considering that the mathematical expectation of small random additions due to the action of noise with a normal distribution is equal to zero, we obtain the following complete system of linear equations with two unknowns $t_{00}^{ + }$ and *T*:

26 $$\left\{ \begin{gathered} M(Y_{1} ) = 2(n + 1)t_{00}^{ + } + \left( {n + 1} \right)\left( {n + \frac{1}{\nu }} \right)T, \hfill \\ M(Y_{2} ) = \left( {n + 1} \right)\left( {n + \frac{1}{\nu }} \right)\,t_{00}^{ + } + \left( {n + 1} \right)\left( {\frac{{3nv + v^{2} n(2n + 1) + 3}}{{3\nu^{3} }}} \right)\,T. \hfill \\ \end{gathered} \right.$$

Having solved the system by Cramer’s method^33^, we obtain the required representation of the period in terms of a linear combination of the first moments of the complete statistic **Y**:

27 $$T = \frac{{6v^{2} }}{{(n + 1)(n^{2} v^{2} + 2nv^{2} + 3)}}\left[ {M(Y_{2} ) - \frac{nv + 1}{{2v}}M(Y_{1} )} \right]$$

Thus, the estimate of the pulse repetition period (27) is presented in the form (16). According to the uniqueness theorem for the effective estimate, the only effective estimate of the pulse repetition period is:

28 $$\hat{T} = \frac{{6v^{2} }}{{(n + 1)(n^{2} v^{2} + 2nv^{2} + 3)}}\left[ {Y_{2} - \frac{nv + 1}{{2v}}Y_{1} } \right]$$

Taking into account the expressions for *Y*_*1*_ and *Y*_*2*_, the effective estimate of a pulses period is:

29 $$\hat{T} = \frac{3v}{{(n + 1)(n^{2} v^{2} + 2nv^{2} + 3)}}\sum\limits_{i = 0}^{n} {\left[ {\left( {2iv - nv - 1} \right)t_{i}^{ + } + \left( {2iv - nv + 1} \right)t_{i}^{ - } } \right]} \,.$$

The variance of the estimate $\hat{T}$ is given by

30 $$\begin{aligned} D(\hat{T}) & = \frac{{\left( {3v} \right)^{2} }}{{(n + 1)^{2} (n^{2} v^{2} + 2nv^{2} + 3)^{2} }}\sum\limits_{i = 0}^{n} {\left[ {\left( {2iv - nv - 1} \right)^{2} D\{ t_{i}^{ + } \} + \left( {2iv - nv + 1} \right)^{2} D\{ t_{i}^{ - } \} } \right]} \\ & = \frac{{\left( {3v} \right)^{2} \sigma_{ + }^{2} }}{{(n + 1)^{2} (n^{2} v^{2} + 2nv^{2} + 3)^{2} }}\sum\limits_{i = 0}^{n} {\left[ {\left( {2iv - nv - 1} \right)^{2} + \left( {2iv - nv + 1} \right)^{2} } \right]} , \\ D(\hat{T}) & = \frac{{6v^{2} }}{{(n + 1)(n^{2} v^{2} + 2nv^{2} + 3)}}\frac{1}{{\left[ {qs^{\prime}(t_{00}^{ + } )} \right]^{2} }} \\ \end{aligned}$$

Here we assume that D {${t}_{i}^{+}$} = D{${t}_{i}^{-}$} = $\sigma_{ + }^{2}$ =  ${1 \mathord{\left/ {\vphantom {1 {\left[ {qs^{\prime}(t_{00}^{ + } )} \right]^{2} }}} \right. \kern-\nulldelimiterspace} {\left[ {qs^{\prime}(t_{00}^{ + } )} \right]^{2} }}$.

In order to exclude the parameter $s^{\prime}(t_{00}^{ + } )$ from the number of a priori unknowns, its estimate in the approximation of a quasi-rectangular pulse shape is obtained. Based on the criterion of maximum likelihood, the variance $D(\hat{T})$ will be minimal for such values of the threshold *H*, at which the steepness of the signal front at the point of intersection is maximum, i.e. we investigate the derivative of the function $s^{\prime}(x)$ to the maximum absolute value. Therefore, we find the second derivative $s^{\prime\prime}(x)$ (31), equate it to zero and obtain Eqs. (32) and (33)

31 $$s^{\prime\prime}(x) = \left\{ \begin{gathered} \frac{{\pi \,k^{2} }}{{4\left( {k - 1} \right)^{4} }}\exp \left\{ { - \frac{\pi }{8}\frac{{\left( {2xk - 1} \right)^{2} }}{{\left( {k - 1} \right)^{2} }}} \right\}\left( {4\pi x^{2} k^{2} - 4\pi xk - 4k^{2} + 8k - 4 + \pi } \right),\quad x > \frac{1}{2k}, \hfill \\ 0\,,\quad \left| x \right| \le \frac{1}{2k}, \hfill \\ \frac{{\pi \,k^{2} }}{{4\left( {k - 1} \right)^{4} }}\exp \left\{ { - \frac{\pi }{8}\frac{{\left( {2xk + 1} \right)^{2} }}{{\left( {k - 1} \right)^{2} }}} \right\}\left( {4\pi x^{2} k^{2} + 4\pi xk - 4k^{2} + 8k - 4 + \pi } \right),\quad x < - \frac{1}{2k}, \hfill \\ \end{gathered} \right.$$

32 $$4\pi x^{2} k^{2} - 4\pi xk - 4k^{2} + 8k - 4 + \pi = 0$$

33 $$4\pi x^{2} k^{2} + 4\pi xk - 4k^{2} + 8k - 4 + \pi = 0$$

Each of these equations has two solutions, but we will only consider roots that are greater than $1/2k$ for (32) and less than $- 1/2k$ for (33) for $k > 1$, since the other roots have no physical meaning. The selected solutions corresponding to Eqs. (32) and (33) have the following form:

34 $$x_{00}^{ + } = \frac{{\pi + 2\sqrt {\pi k^{2} - 2\pi k + \pi } }}{2\pi k} \,$$

35 $$x_{00}^{ - } = \frac{{ - \pi - 2\sqrt {\pi k^{2} - 2\pi k + \pi } }}{2\pi k} \,$$

The derivative of the dimensionless signal $s^{\prime}(x)$ takes the minimum value at *x* = *x*_*00*_^+^, and the maximum value at *x* = *x*_*00*_^*-*^, but in absolute value they are equal and maximum

36 $$\left| {s^{\prime}(x_{00}^{ + } )} \right| = \left| {s^{\prime}(x_{00}^{ - } )} \right| = \frac{\sqrt \pi k}{{\sqrt e (k - 1)}} \,$$

The physical meaning of the obtained variables can be stated as follows: *x*_*00*_^+^—the initial phase of the quasi-rectangular signal *s(x)*, i.e. the first crossing of the *H* level by the signal leading edge, at which the variance of the signal period estimate $\hat{T}$ reaches a minimum value; *x*_*00*_^*−*^—similarly, only for the trailing edge.

By inverting the variables t = □0x, we pass to the signal s(t), where t is no longer a dimensionless quantity, and time has the dimension of time. Then the desired values *t*_*00*_^+^ and *s’(t*_*00*_^+^*)* will take the following form:

37 $$t_{00}^{ + } = \tau_{0} x_{00}^{ + } = \tau_{0} \frac{{\pi + 2\sqrt {\pi k^{2} - 2\pi k + \pi } }}{2\pi k} \,$$

38 $$\left| {s^{\prime}(t_{00}^{ + } )} \right| = \frac{\sqrt \pi k}{{\tau_{0} \sqrt e (k - 1)}} \,$$

Taking into account the assumption of the signal off-duty factor ν = T/τ_0_ = 2, we present s’(t_00_^+^) in terms of the period:

39 $$\left| {s^{\prime}(t_{00}^{ + } )} \right| = \frac{2\sqrt \pi k}{{T\sqrt e (k - 1)}} \,$$

Substituting the obtained derivative value and the signal off-duty factor value, ν  = 2, in (30), for the variance of the signal period estimate, we obtain:

40 $$D(\hat{T}) = \frac{6}{{(n + 1)(4n^{2} + 8n + 3)}}\frac{{eT^{2} \left( {k - 1} \right)^{2} }}{{q^{2} \pi \;k^{2} }}$$

## Comparison and analysis of methods

We find the relative error in estimating pulse repetition period obtained by the method of complete sufficient statistics. The relative error for n measurements is determined as follows:

41 $$\delta_{1} = \frac{{\sqrt {D(\hat{T})} }}{T} = \frac{{\sqrt {6e} }}{{\sqrt {\pi \;(n + 1)(4n^{2} + 8n + 3)} }}\frac{{\left( {k - 1} \right)}}{q\,k}$$

Consider the relative error δ_0_, which depends on the signal-to-noise ratio q and the number of measurements n. Assume that the squareness coefficient of the pulse k is 1.2 (k = 1.2) In practice, a differential scheme may be used to estimate k, which allows to conclude about the steepness of the leading and trailing edges of the pulse:

42 $$\delta_{1} = \frac{\sqrt e }{{q\sqrt {6\pi \;(n + 1)(4n^{2} + 8n + 3)} }}$$

**Figure 2:**
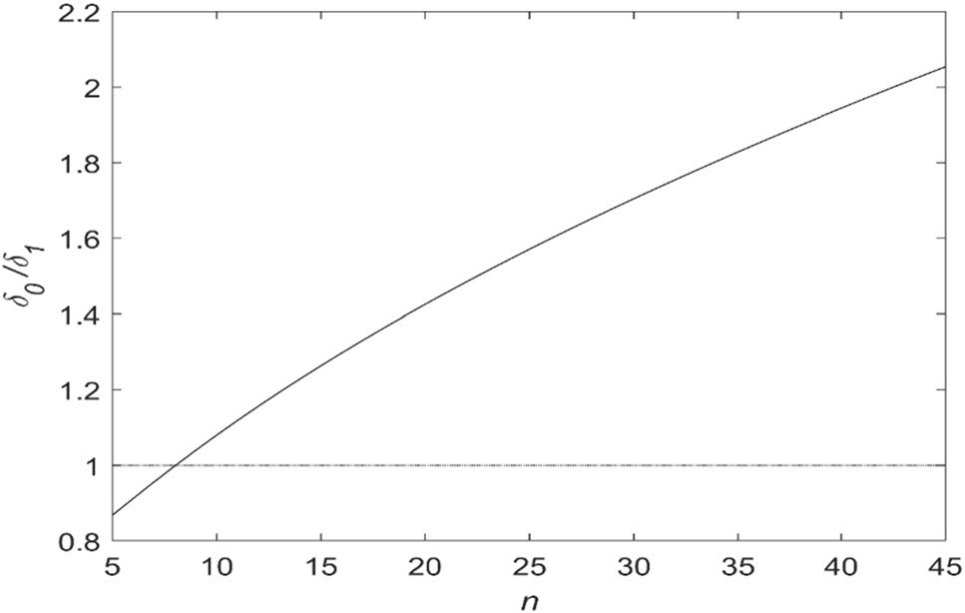
The ratio of relative pulse period determination errors δ_0_/δ_1_, where δ_0_ and δ_1_ are relative errors of the MLE and CSS methods respectively.

Figure 2 shows the ratio of the relative errors δ_0_ / δ_1_ obtained by the MLE δ_0_ and CSS δ_1_, calculated using formulas (11) and (42) for a different number of pulses in the sample n. The figure shows that this ratio is greater than 1 for n > 8 and increases with rise in the sample size. This indicates that the estimate obtained by CSS method is more efficient than the MLE estimate for n > 8.

**Figure 3:**
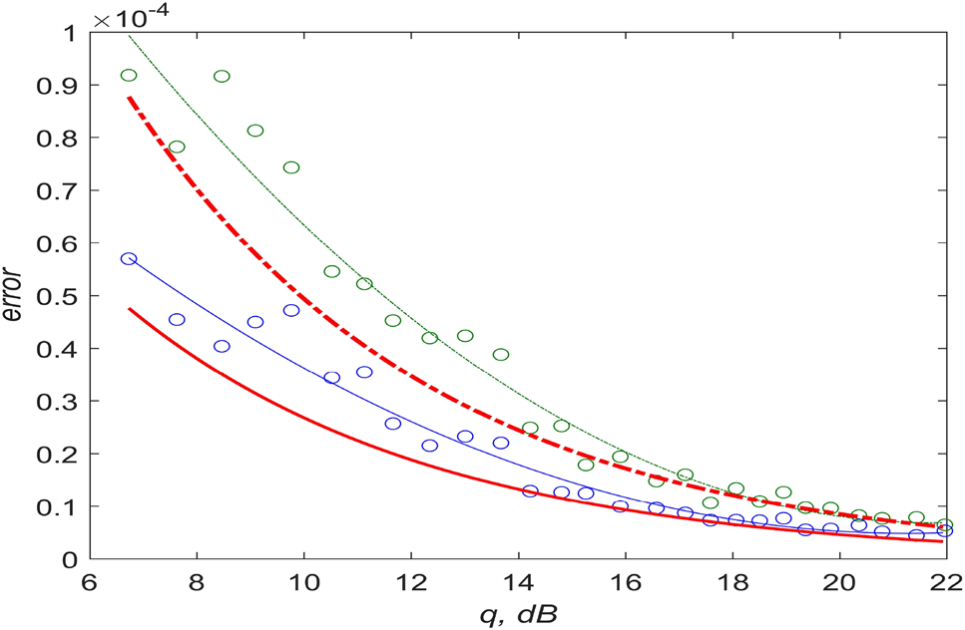
Relative errors in measuring the pulse repetition period: dotted lines correspond to the MLE, solid lines correspond to the CSS method, bold lines are errors calculated by formulas (11) and (42), thin lines are results of least squares method approximations of numerical modeling (circles—results of numerical modeling). The signal-to-noise ratio (SNR) is plotted along the X axis.

The greatest gain from using the CSS method can be obtained at low values of the signal-to-noise ratio, when the accuracy of both methods is low. Let us fix the number of measurements (for example, n = 16) to estimate the energy gain of the CSS methodology in comparison with traditional approaches for the finite and small sample size. The dependencies $\delta_{0}$ and $\delta_{1}$ on *q* in the range of 0–10 dB will give a difference in the achieved signal-to-noise ratio of the order of 2 dB in favor of an estimate using the CSS methodology (Fig. 3).

## Numerical modeling, results and discussion

Expressions (11) and (42) only allow us to theoretically estimate the energy gain of the CSS method in comparison with the MLE when solving the problem of estimating the pulse repetition period. At the same time, to verify such estimates, numerical simulations were performed in the MATLAB environment. For this purpose, a sequence of pulses were generated against a background of additive white Gaussian noise with intra-pulse quadrature phase shift keying (QPSK). The order of occurrence of the pulse at a given time position and its carrier frequency in the receiving band were determined by a pseudo-random sequence generator.

Two alternative algorithms for estimating the pulse repetition period were applied to the resulting implementation: based on the MLE and CSS methods, computed according to the formulas (12) and (29). The number of pulses in the implementation n ranged from 10 to 50, and the signal-to-noise ratio q ranged from 6 to 22 dB.

The results of the estimation for n = 16 as a function of q are shown in Fig. 3. The relative errors of the two alternative algorithms are presented. It is easy to see that the energy gain of the CSS method is more than 2 dB in the q interval up to 12 dB. The difference is reduced to 1 dB with an increase in the signal-to-noise ratio q, while it remains that the traditional estimate asymptotically tends to the unique effective one when $q \to \infty$.

In addition, numerical modeling demonstrated the stability of the algorithm. The algorithm effectively estimates the pulse repetition period using the CSS methodology with respect to variations in the threshold level H (red line in Fig. 1) over a wide range (up to 10 dB). With the same variation, the estimate synthesized on the basis of the MLE approach is not stable.

Let us compare the computational costs required to find the pulse repetition period using the MLE and CSS methods. Both methods are linear with respect to the sample size of signal pulses (they belong to the complexity class O (n)). In the case of the traditional estimation of the pulse repetition period, obtained by the MLE method according to formula (12), the number of addition/subtraction $C_{ + , - }^{MLE}$, multiplication $C_{*}^{MLE}$ and division $C_{/}^{MLE}$ operations after optimization depends on the number of pulses in the sample as follows

43 $$C_{ + , - }^{MLE} \left( n \right) = 3n + 1,\;\;\;\;C_{*}^{MLE} \left( n \right) = 0,\;\;\;\;C_{/}^{MLE} \left( n \right) = 1 + n$$

In the case of the pulse repetition period estimation obtained by the CSS method according to formula (27), the number of operations of addition/subtraction $C_{ + , - }^{CSS}$, multiplication $C_{*}^{CSS}$ and division $C_{/}^{CSS}$ after optimization is

44 $$C_{ + , - }^{CSS} \left( n \right) = 3\left( {n + 1} \right) + 5,\;\;\;C_{*}^{CSS} \left( n \right) = 2\left( {n + 1} \right) + 6,\;\;\;\;C_{/}^{CSS} \left( n \right) = 1$$

Comparing (43) and (44), we conclude that the CSS method requires more operations, however, additional optimization of expression (29) for a specific processor architecture or logical cell array (LCA) is possible, examples of which are described in^34,35^. This optimization is a separate engineering problem and is beyond the scope of this work.

## Conclusion

The methodology of complete sufficient statistics is applied to solve the problem of finding an effective estimate of the pulse repetition period. Taking into account the probability distribution functions, a mathematical apparatus is constructed that allows using the Lehmann–Scheffe theorem and its corollary. An analytical expression for the effective estimation of the pulse repetition period is obtained.

Expressions for the variance and relative error of the pulse repetition period estimation are obtained using the CSS methodology depending on the duty-off-factor, squareness coefficient, signal-to-noise ratio and sample size. In the framework of the estimation theory, the lower bound of the accuracy of the estimation of the pulse repetition period is calculated. The relative errors of estimates are compared using traditional methods of MLE and the CSS methodology.

It is shown that for a finite and small value of the sample size (n = 16) in the range of signal-to-noise ratios of 5–12 dB, the relative root-mean-square error of estimates for the pulse repetition period by CSS method is significantly less than the error of the traditional estimate by MLE method.

The results obtained have a wide range of applicability and can be used in the design of control and measuring equipment in the oil and gas industry, in the development of medical equipment, in the field of telecommunications, in the design of pulse-Doppler radars, etc.

## Data Availability

The datasets generated during and/or analyzed during the current study are available from the first author on reasonable request.
